# Current Advances in Immunological Studies on the Vespidae Venom Antigen 5: Therapeutic and Prophylaxis to Hypersensitivity Responses

**DOI:** 10.3390/toxins10080305

**Published:** 2018-07-24

**Authors:** Murilo Luiz Bazon, Lais Helena Silveira, Patricia Ucelli Simioni, Márcia Regina Brochetto-Braga

**Affiliations:** 1 Laboratório de Biologia Molecular de Artrópodes-LBMA-IB-RC-UNESP (Univ Estadual Paulista), Av. 24-A, n_ 1515, Bela Vista, Rio Claro 13506-900, SP, Brazil; bazonmurilo@gmail.com (M.L.B.); lenapascon@gmail.com (L.H.S.); 2 Departamento de Biomedicina, Faculdade de Americana, FAM, Av. Joaquim Bôer, 733 Jardim Luciane, Americana 13477-360, SP, Brazil; psimioni@gmail.com or patriciasimioni@fam.br; 3 Centro de Estudos de Venenos e Animais Peçonhentos-CEVAP (Univ Estadual Paulista), Rua José Barbosa de Barros, 1780, Fazenda Experimental Lageado, Botucatu 18610-307, SP, Brazil

**Keywords:** Antigen 5, Hymenoptera, immune response, hypersensitivity, anaphylaxis

## Abstract

Although systemic reactions caused by allergenic proteins present in venoms affect a small part of the world population, Hymenoptera stings are among the main causes of immediate hypersensitivity responses, with risk of anaphylactic shock. In the attempt to obtain therapeutic treatments and prophylaxis to hypersensitivity responses, interest in the molecular characterization of these allergens has grown in the scientific community due to the promising results obtained in immunological and clinical studies. The present review provides an update on the knowledge regarding the immune response and the therapeutic potential of Antigen 5 derived from Hymenoptera venom. The results confirm that the identification and topology of epitopes, associated with molecular regions that interact with antibodies, are crucial to the improvement of hypersensitivity diagnostic methods.

## 1. Introduction

The order Hymenoptera (Apocrita, Aculeata) is the main group among insects, which consists of three main families: Vespidae (comprising the best-known eusocial wasps from genera *Polistes*, *Polybia*, *Vespa*, *Vespula* and *Dolichovesvula*) [1,2,3], Formicidae (ants) and Apidae (bees). Social wasps are responsible for the increasing number of accidents recorded. In southeastern Brazil, *Polybia paulista* is one of the most relevant social wasp from the medical point of view, justifying the need for a more detailed characterization of the action mechanisms of the major proteins and allergenic compounds of its venom. Although the anaphylactic shock associated with immediate hypersensitivity reactions caused by allergenic proteins present in venoms affects a small part of the world population, Hymenoptera stings are among the main causes of systemic allergic responses [4,5], representing 9 to 23% of hypersensitivity reactions [5,6].

The classical symptoms of stings are local burning, followed by edema and pain that can last for long periods. In addition, respiratory and circulatory reactions are common in allergic conditions [4,5]. The amount of venom to which the person has been exposed and the level of individual sensitivity to allergens should be taken into account while evaluating the immunological and clinical response [6,7,8]. The allergen components of the venom trigger the immune system response by producing specific antibodies [9]. In more severe stages, the immune response can lead to intense systemic inflammatory processes and fatal anaphylaxis reactions [4,5,6].

The molecular characterization of these allergens has brought promising results in clinical and immunological studies, supporting the development of therapeutic treatments and the prophylaxis of hypersensitivity responses. In addition, such knowledge can provide a better understanding of the allergic processes and allow the identification of epitopes and molecular regions interacting with antibodies [6,8,10].

### Venom

Eventual stings caused by Hymenoptera account for 20 to 40% of all types of anaphylaxis reported per year [11,12]. These venoms are composed of a complex mixture of proteins, enzymes, biologically active peptides and low molecular weight molecules, which are responsible for prolonged pain, edema, erythema, and allergic and systemic reactions [13,14]. Systemic reactions occur mainly in allergic patients, and clinical symptoms include generalized urticaria, angioedema, blood pressure drop, bronchospasm, cardiac arrest, and respiratory and anaphylactic shock [12,15,16,17].

Allergy diagnosis includes the history of a systemic reaction, a positive response to the skin test and the detection of venom-specific IgE antibodies. However, correct diagnosis is not always easy due to problems and limitations of both tests, especially when it is not possible to identify the species [18,19]. Moreover, allergic patients may present positive reactions to more than one insect venom of the Hymenoptera order, which may be caused by the cross reactivity of one or more allergens, considering the similarity between their primary sequences [12,15,16,17,20,21,22,23,24]. False-positive results can also be observed due to the cross-reactivity of patient IgE against the cross-reactive carbohydrate determinants (CCDs) present in most Hymenoptera venom allergens [23,24,25,26,27]. Having these difficulties solved and the correct diagnosis, immunotherapy with the whole venom of culprit insect represents the most effective treatment to reduce the risk of subsequent systemic reactions.

Wasp venom contains a variety of proteins such as phospholipases, hyaluronidases, Antigen 5, phosphatases, and serine proteases. Phospholipases have been chemically characterized in *Apis melífera* [28], venoms of neotropical wasps and ants [29,30,31,32,33], being responsible for the hydrolysis of the plasma membrane phospholipids, allowing the diffusion of some toxins into the cells. Phospholipase is also responsible for the formation of edema [34]. Hyaluronidase is a 45 kDa glycoprotein [35] that hydrolyzes hyaluronic acid, a polysaccharide of high molecular mass located in the cellular interstice, with the property of maintaining cell adhesion. By the action of hyaluronidase, hyaluronic acid is transformed into small fragments, significantly reducing its viscosity and facilitating the diffusion of the venom components into the cells [36,37]. Phosphatases are found in large quantities in bee venom. These enzymes act as important allergens [38]. The venom of the wasp *P. paulista*, presents two types of phosphatase activity: acid and alkaline [32]. The esterases are important in the cell lysis process; however, its specific function has not been defined [38].

Social insect venoms do not contain significant amounts of proteases [39,40,41]. However, high protease activity has already been observed in venoms of the social wasp *Polistes infuscatus*, in *Eciton burchelli* ants and *Bombus pennsylvanicus* bees [38,42], and some protease activity was found in venoms of Hymenoptera: Vespidae, commonly found in São Paulo State [43]. Proteases catalyze the breakdown of peptide bonds into proteins, and the term “peptidases” may be used to denote any enzyme that hydrolyzes this type of linkage [44].

## 2. Antigen 5

King et al. (1978) identified a protein of approximately 23 kDa of *Dolichovespula maculata* venom, naming it Antigen 5 (Ag 5) [45]. Since then, Antigen 5 has been frequently reported to be the most allergenic venom component in different species of social wasps, such as those belonging to the genus *Dolichovespula*, *Vespa*, *Vespula*, *Polistes* and *Polybia* [46,47,48,49,50,51]. Interestingly, Ag 5 also exhibits sequence homology with other proteins from various tissues, such as ant venoms, tomato leaf tobacco, mammalian testis proteins and human brain tumor [52].

This antigen belongs to a superfamily composed of proteins that are rich in cysteine ​​residues (CRISP-Cysteine-Rich Secretory Proteins). Analysis of its domains have showed that it belongs to a CRISP subgroup, antigen 5 and Pr-1 (CAP) [52]. Ag 5-related proteins are also found in glial cell tumors [53]. According to Milne et al. (2003), Ag 5 is a protein that presents a high similarity of sequence with the protease of the venom of the family Conidae [54]. However, its biological function remains unknown and there is no knowledge of its biological action as a component of the venom of Vespidae family [47]. Animal studies have shown that, despite having no toxic action, Ag 5 may be associated with hypersensitivity responses [10].

### 2.1. Superfamily CAP

The superfamily CAP [cysteine-rich secretory proteins (CRISPs), Ag 5 and pathogenesis-1 (PR-1) related proteins] was named after the recognition of sequence similarity between CRISPs in eukaryotes, reptile venoms, plant pathogenic defense proteins or other stress responses (PR, PR-1) as found in tobacco leaf and tomato (P14-A-PRPs), CRISP and mammalian reproductive organ (TPX-1) specific proteins. CAP comprises three domains: N-terminal PR domain, a hinge region and a cysteine-rich C-terminal domain [55]. Evidence suggests that CAPs plays an important role in the reproductive function, immune system, tumors and chronic diseases, organogenesis, and development of mammals. Asojo et al. (2005) reported a high similarity in the primary sequence and three-dimensional structure of the Na-ASP-2 protein (from CRISP-1 family and the one present in the nematode parasite *Necator americanus*) with Ag 5 of wasp venom. The potential of this protein to be used in the development of vaccines containing blocking or ligand antibodies to disrupt the cellular activation responses has already been demonstrated [52,56].

### 2.2. Isoforms of Ag 5

**Figure 1:**
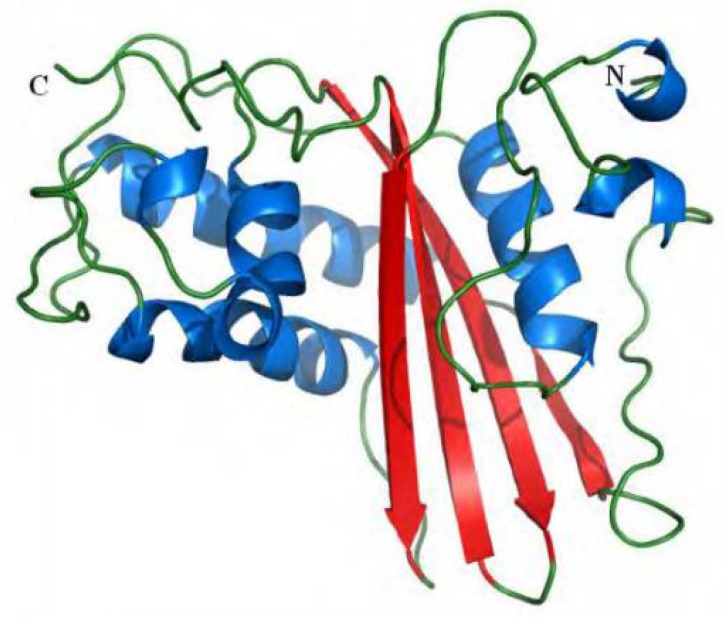
Three-dimensional molecular model of the antigen 5 allergen from social wasp *Polybia paulista* venom [60]. Reprinted with permission from [60], 2014, American Chemical Society.

Antigen 5 isolated and structurally characterized from the venom of the wasp *Polybia scutellaris rioplatensis* [49,57,58] has 207 amino acid residues, eight cysteine-rich residues forming four disulfide bonds, molecular mass around 23 kDa and isoelectric point around nine [36,47,59]. The three-dimensional structure of venom Ag 5 of *Vespula vulgaris* was determined by X-ray crystallography, revealing that it has a secondary structure composed of five α-helices and four β-sheets [59]. In proteomic studies, six isoforms of the Ag 5 of *P. paulista* have been identified [36]. The most abundant isoform has been extensively analyzed through mass spectrophotometry, and several of its post-translational modifications have been determined. Its structural model (Figure 1) showed three α-helices, one helix 3_10_, and four β-sheets covering 28% and 17%, 9% of its sequence. Linear epitopes of this form have also been identified, mapped and immunologically characterized [48,60].

Antigen 5 from the venoms of *Polybia paulista* (Poly p 5) and of *P. scutellaris rioplatensis* presented a high similarity (59.3–93.7%) with its counterpart in the other Vespidae venom. Considering the diversity of substances in venoms and the scarce knowledge on the immunological potential of their allergenic components, such as the Ag 5 protein, further studies are needed to elucidate the processes involved in sensitization and allergic response [61]. The in-depth knowledge on the antigen-directed immune response presented in the venom of Hymenoptera may increase therapeutic possibilities for hypersensitive patients.

### 2.3. Immune Response to Allergens

The immune responses to wasp venoms are complex and can include systemic allergic/hypersensitivity disorders [62]. In general, the wasp venom leads to a type I hypersensitivity reaction [5,8,10]. The antigens induce a helper (Th) 2 T lymphocyte cellular response profile, characterized by the production of specific IgE antibodies, as well as the secretion of interleukins (IL) -4 and IL-5 [63,64]. In the immune response, interferon gamma (IFN-γ), a proinflammatory cytokine, stimulates proinflammatory gene expression, such as the inducible synthase genes (iNOS) and cyclooxygenase-2 (COX-2). Macrophages, depending on the microenvironment, can differentiate into distinct types: classically activated macrophages (M1) and alternatively activated macrophages (M2) with anti-inflammatory profile [65,66]. The inducible isoform of nitric oxide synthase (iNOS) stimulates the production of nitric oxide (NO) from l-arginine [67], one of the functions of macrophages M1 [68]. Cytokines IL1, IL6, IL-12, and TNF-α are known to have inflammatory potential, whereas IL-10 and TGF-β act in the modulation and inhibition of the immune response [69]. The gene encoding the iNOS enzyme is controlled by NF-κB, which plays a key role in inflammatory and immune cell responses [70]. The NF-κB transcription complex is present in the cytoplasm, bound to inhibitory proteins called IκB, maintaining them in the inactive form. Inhibition of NF-κB is associated with inflammatory diseases and can be a potential therapeutic target [71].

As noted above, exposure to this class of venom insect triggers a type I hypersensitivity reaction. IL-4 induces CD4 + T to differentiate into Th2, crucial for the entire development of the hypersensitivity framework. Immunotherapies for this type of response aim to increase suppressor cytokines, such as IL-10 and TNF-β and decrease IL-4 secretion [72].

### 2.4. Cross Reactivity

One of the main obstacles to find an effective treatment for hypersensitivity responses is the occurrence of unspecific or undefined reactions, i.e., immune cross reactivity, a consequence of the significant similarity between the primary sequences of the allergenic proteins [50] and the presence of cross-reactive carbohydrate determinants (CCDs) of N-linked glycans [73,74,75] in some Hymenoptera venom allergens [76,77].

Generally, the diagnostic of allergic response is based on the patient’s clinical history, detection of specific IgE on the skin and/or blood of the allergic individual. When usual analyses are not conclusive, basophil activation or histamine releasing tests are performed to identify the culprit venom [26]. However, false-negative responses may occur due to the low amount of IgE detected or the low level of sensitivity of the test applied. False-positive responses can be caused by cross-reactivity with allergens from different venoms, whose epitopes have similar conformations [78,79].

The high similarity between the primary sequences of the allergens of several species of social wasps promotes a wide potential for the occurrence of cross reactivity between the different species. The similarity between Ag 5 from different social wasp species could explain the broad cross-reactivity between proteins. Posttranslational modifications (PTMS) of different species, such as glycosylation, could also be a cause of cross-reactivity of the Hymenoptera venom [47,60]. The Ag 5 allergen demonstrated cross reactivity with the venom of other species as *Agelaia pallipes* and *Apis mellifera,* being immunoreactive in the experiments performed [51,80,81]. The presence of IgE against carbohydrate-determining regions (CCD) occurred in more than 80% of the samples positively tested for both species [82].

Studies on the primary structure and immunological response of the Ag 5 from venom from wasp species (endemic in the northern hemisphere) have reported that the identity of Ag 5 sequences in species of the same genus is approximately 98%, whereas among the different genera, such as *Vespula* and *Polistes*, this value is approximately 57% (Figure 1) [55]. According to the allergen list from the International Union of Immunological Societies (IUIS), Ag 5 is present in venoms of almost all species of the genus belonging to family Vespidae, including *Solenopsis* ants (*Solenopsis invicta*, Sol i 3; *Solenopsis richteri*, Sol r 3; *Solenopsis saevissima*, Sol s 3), whose allergens have high similarity to Ag 5 from other Vespidae venoms [83].

Nevertheless, the IgE associated with the cross-activity between Ag 5 of *V. vulgaris* and Sol i 3 from *Solenopsis invicta*, which show 44% similarity on amino acid sequence [55], has not been investigated. The fact that the Ag 5 homologous proteins found in some ant venoms do not exhibit cross-reactive antigen reactivity with the same protein in vespid venoms is a consistent result regarding the low degree of structure conservation and the length of the loops in these allergens. However, some cross reactivity has been observed between Ag 5 of vespids and the homologous proteins (from CRISP family) of other animals [53]. Müller et al. [84] observed cross reactivity in human serum between *V. vulgaris* Ag 5 venom allergen and mammalian testis proteins belonging to the family of cysteine-rich secretory proteins (hCRIsp).

Van Vaerenbergh et al. [18] demonstrated the expression of a molecule similar to wasp venom Ag 5 of *Apis mellifera* bee venom and named apidaecina. In addition, the authors reported that this molecule is expressed in different tissue types, such as the hypopharyngeal, brain, and midgut glands, more abundantly in the brain. The comparative analysis of this sequence was clearly paralleled to the sequences of Ag 5 already described for wasp (*Vespula*, *Vespa*, *Dolichovespula*, *Polistes*, *Polybia* and *Rhynchium*) and ant (*Pachycondyla* and *Solenopsis*) venoms [18,83].

Comparing the primary sequences of Dol m 5 with those of the defense proteins related to the pathogenicity of these viruses in plants, 28% of conservation was found. When consuming tomato products or smoking, these patients developed antibodies against PRPs, which in turn showed cross reactivity with Dol m 5 of *Dolichovespula maculate* [59,85].

Despite this high similarity between the Ag 5 proteins of the *P. paulista* and *P. scutellaris* venoms, the component was described as a hypoallergenic molecule in *P. scutellaris* [44,65]. This result is conflicting with studies on *P. paulista* [34,35] and other wasps, such as *Vespula vulgaris* (Ves v 5) [58,86,87,88]. Antigen 5 from *P. scutellaris* was reported as a variant with reduced reactivity to specific IgE and anaphylactic activity and so, it was considered an important allergen to be used in immunotherapy of allergic patients [49]. Thus, the importance of cross-reactivity among insect venoms in clinical practice is unquestionable, since these interactions have a direct impact on the diagnosis and on the definition of the best therapeutic approach. Using recombinant Ag 5, immunologically and structurally fully characterized, from seven allergy-relevant species from Vespoidea group, Schiener et al. [16,20] investigated the immunological IgE cross-reactivity through ImmunoCAP, ELISA, cross-inhibition and basophil activation test (BAT). They concluded that Ag 5 is not an appropriate diagnostic marker for vespid venom, since high levels of cross-reactivity were observed in many of the analyses performed. Therefore, the investigation of new Ag 5 variants in other vespid species and the development of more accurate methodologies may assist the precise diagnostic of wasp venom allergies.

### 2.5. Potential of the Molecule Ag 5 in Immunotherapy

Considering the evolutionary diversity of proteins from CAP superfamily, several functional relations have been proposed for them, such as the regulation of the immune system [52]. Ag 5 is part of the salivary proteins that supposedly function in the suppression of the host immune system or in the prevention of coagulation [89,90,91]. Ag 5 has been demonstrated to trigger immune and inflammatory responses via mast cell activation. This activation occurs when molecules associate with G-protein receptors through different mechanisms: (a) when polycationic peptides bind to the G-protein receptors present in cell membranes and βγ subunits stimulate phospholipase C, leading to the release of mediators [92] or (b) by the cross-linking of IgE receptors on the cell membrane. This results in the aggregation of high affinity receptors FcεRI and the secretion of substances that may have effector, immunoregulatory or autocrine actions.

Mast cell activation generates three types of biological responses: secretion of preformed granule contents through a regulated process of exocytosis, synthesis and secretion of lipid mediators, and synthesis and secretion of cytokines. These responses occur due to FcεRI cross-linking, which initiates a signaling cascade in the mast cells, involving protein tyrosine kinases and leading to the release of mediators [93,94].

The evidence of the biological function of the Ag 5 is related to the poor inhibition of trypsin in human glioblastoma cells [95]. Trypsin inhibition has not been reported for other species of wasps and bees. Venom Ag 5 from several wasp species has been expressed as recombinant proteins in both prokaryotic and eukaryotic systems [87]. Thus, expression of the Ag 5 allergen has the potential to provide a large number molecule for diagnosis and therapy. In general, recombinant allergens have been considered a promising alternative for the improvement of specific allergen immunotherapy and in in vitro diagnosis of allergic sensitization [96].

Bohle et al. [97] provided evidences that the T-cell immune response to Hymenoptera allergens differs from the typical Th2-dominated response observed to the most inhalant allergens. Their report showed that the immune response to Ag 5 involves high secretion of IL-4 and low levels of IFN-gamma. Surprisingly, the secretion of IL-10, which commonly is associated with the suppression of allergen-specific T cell responses in healthy individuals, had no differences between Ves v 5-specific T cell clones from allergic and non-allergic individuals [97].

Antigens 5 is an inappropriate marker for differential IgE diagnostics in vespid venom allergy since it can cause extensive cross-reactivity in various diagnostic settings [20]. However, there are few reports related with immunotherapies with Ag 5 in use [15,16,58]. Although the European guidelines still recommend the venom immunotherapy with *Vespula* venom in order to achieve an adequate protection against *Vespa crabro* venom, studies with Antigen 5 showed that sensitization may occur. Immunotherapy, when available, is still the safest method, since venom extracts are available [98].

## 3. Future Perspectives

Ag 5 is a common allergen found in social wasp venoms and in many different animal and plant systems. Immunotherapy with venom extracts from social insects is highly effective and widely used in the treatment of patients with a history of anaphylaxis [87]. Specific immunotherapy is the only treatment for type I allergies and is based on the accurate allergy history of the individual and the results of skin and RAST (Radioallergosorbent) diagnoses, which confirm the presence of IgE [99,100]. The risk of inefficiency of specific immunotherapy can be associated with *de novo* sensitization to new allergenic proteins (or cross-reacting allergens) to which patient had not presented any reaction [19]. On the other hand, patients presenting systemic reactions and opposed immunotherapy, lost sensitivity in the same proportion as those who underwent treatment [101]. One way to reduce the risk of anaphylaxis during specific immunotherapy is to use modified allergens, with decreased interaction [102]. Another possibility is the preparation of genetically modified allergens or peptide allergenic derivatives with reduced allergenic activity that will induce a specific interaction of the allergen based on IgG antibodies [103]. Although antigen 5 function has not been clarified, the molecule is a strong candidate to be used in immunotherapy in patients allergic to social wasp venom. It has been demonstrated that the soluble recombinant form of Poly p 5 (rPoly p 5) obtained through expression in *P. pastoris* is allergenic and induces an immune response that occurs qualitatively at the same level as its natural variant (nPoly p 5) [104]. This finding strongly indicates that this molecule can be effectively used for the molecular diagnosis of allergies. Studies on the pro-or anti-inflammatory potential of Ag 5, specifically on its ability to stimulate nitric oxide production or cytokine secretion, may be of great relevance for the comprehension of the immunomodulatory potential of this protein.

## Figures and Tables

**Figure 2:**
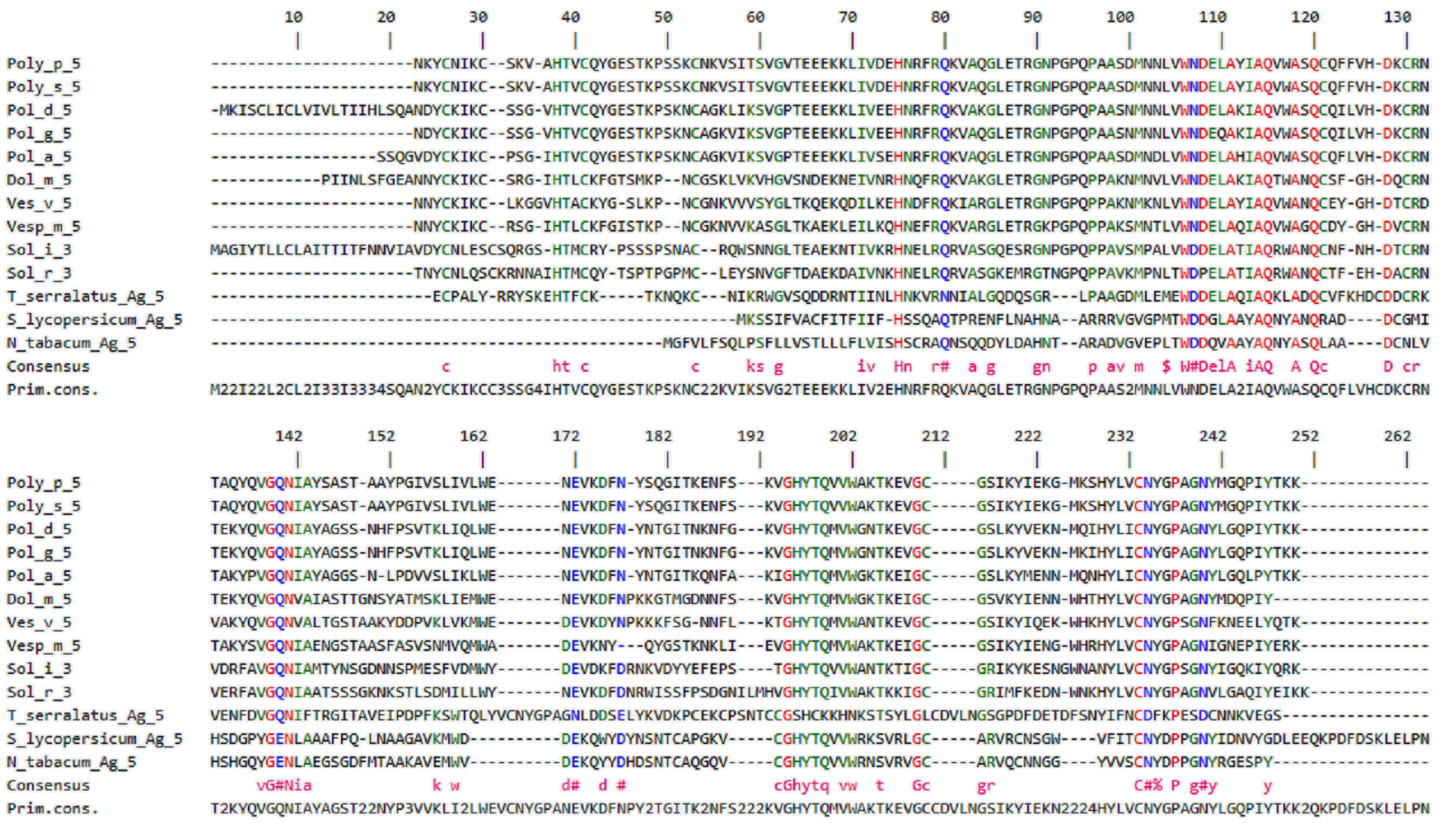
Multiple alignment of primary sequences of venom allergen Ag 5 of wasps (*Polybia*, *Polistes*, *Dolichovespula*, *Vespula* and *Vespa*) with sequences from other members of the superfamily CAP (*Solenopsis invicta*, *Solenopsis richteri*, *Tityus serrulatus*, *Solanum lycopersicum* and *Nicotiana tabacum*). Data referenced in GenBank and adapted.
